# *BMP4* and *PHLDA1* are plausible drug-targetable candidate genes for *KRAS* G12A-, G12D-, and G12V-driven colorectal cancer

**DOI:** 10.1007/s11010-021-04172-8

**Published:** 2021-05-12

**Authors:** Shumpei Ohnami, Kouji Maruyama, Kai Chen, Yu Takahashi, Keiichi Hatakeyama, Keiichi Ohshima, Yuji Shimoda, Ai Sakai, Fukumi Kamada, Sou Nakatani, Akane Naruoka, Sumiko Ohnami, Masatoshi Kusuhara, Yasuto Akiyama, Hiroyasu Kagawa, Akio Shiomi, Takeshi Nagashima, Kenichi Urakami, Ken Yamaguchi

**Affiliations:** 1 Cancer Diagnostics Research Division, Shizuoka Cancer Center Research Institute, 1007 Shimonagakubo, Nagaizumi-cho, Sunto-gun, Shizuoka 411-8777 Japan; 2 Experimental Animal Facility, Shizuoka Cancer Center Research Institute, Shizuoka, Japan; 3 Division of Colon and Rectal Surgery, Shizuoka Cancer Center Hospital, Shizuoka, Japan; 4 Medical Genetics Division, Shizuoka Cancer Center Research Institute, Shizuoka, Japan; 5 SRL Inc, Tokyo, Japan; 6 Drug Discovery and Development Division, Shizuoka Cancer Center Research Institute, Shizuoka, Japan; 7 Immunotherapy Division, Shizuoka Cancer Center Research Institute, Shizuoka, Japan; 8 Shizuoka Cancer Center Research Institute, Shizuoka, Japan; 9 Shizuoka Cancer Center, Shizuoka, Japan

**Keywords:** *BMP4*, *PHLDA1*, Colorectal cancer, *KRAS* mutation, Therapeutic targets

## Abstract

Despite the frequent detection of *KRAS* driver mutations in patients with colorectal cancer (CRC), no effective treatments that target mutant KRAS proteins have been introduced into clinical practice. In this study, we identified potential effector molecules, based on differences in gene expression between CRC patients carrying wild-type *KRAS* (*n* = 390) and those carrying *KRAS* mutations in codon 12 (*n* = 240). CRC patients with wild-type *KRAS* harboring mutations in *HRAS*, *NRAS*, *PIK3CA*, *PIK3CD*, *PIK3CG*, *RALGDS*, *BRAF,* or *ARAF* were excluded from the analysis. At least 11 promising candidate molecules showed greater than two-fold change between the *KRAS* G12 mutant and wild-type and had a Benjamini-Hochberg-adjusted *P* value of less than 1E-08, evidence of significantly differential expression between these two groups. Among these 11 genes examined in cell lines transfected with *KRAS* G12 mutants, *BMP4*, *PHLDA1,* and *GJB5* showed significantly higher expression level in *KRAS* G12A, G12D, and G12V transfected cells than in the wild-type transfected cells. We expect that this study will lead to the development of novel treatments that target signaling molecules functioning with *KRAS* G12-driven CRC.

**Supplementary Information:**

The online version contains supplementary material available at 10.1007/s11010-021-04172-8.

## Introduction

The incidence and mortality rates of colorectal cancer (CRC) have recently been increasing in Japan [1]. Surgical resections can cure CRC in the early stage, and advances in pharmacotherapy have also improved the treatment outcomes in patients with unresectable and advanced/recurrent-stage CRC. However, the five-year survival rate in patients with advanced stage IV CRC is quite low at approximately 18% [2]. Therefore, new therapeutic drugs, particularly molecular targeted agents with fewer adverse drug reactions, need to be developed for improving the prognosis in CRC patients [3]. Advanced CRC is typically treated with monoclonal antibodies targeting epidermal growth factor receptor (EGFR), such as cetuximab or panitumumab, used alone or in combination with standard chemotherapy, but CRC patients harboring *KRAS* mutations do not respond to the antibody-based anti-EGFR treatment [4].

RAS proteins, including KRAS as one of the molecules that play a central role in intracellular signaling pathways, appear to be involved in a wide range of processes including cell proliferation, differentiation, metabolism, and cell death [5–7]. Therefore, drugs that directly target RAS proteins that are ubiquitously expressed as house-keeping genes are more likely to have unanticipated reactions with other proteins in the body [8]. Wild-type *KRAS* has been shown to act as a tumor suppressor gene during the differentiation of myeloid cells [9] and inhibit lung carcinogenesis in murine teratomas [10]. Literature surveys suggest that the wild-type *KRAS* could play an onco-suppressor role [11–13]. *KRAS* mutations are observed in approximately 40% of patients with CRC and occur frequently in codon 12 or 13 and less frequently in codons 146 or 61. A study focusing on immortalized human bronchial epithelial cells reported differences in the degree of constitutive activation of the KRAS protein, rates of increase in tumor cell proliferation, and the degree of activation of proliferative signals downstream of KRAS, depending on the mutation sites in the *KRAS* gene [14]. In addition, downstream effector molecules of KRAS signaling pathways were shown to differ according to tumor type [15]. These observations raised the possibility that the mechanism by which activated KRAS binds preferentially to its downstream partners’ genes, and how these interactions after cell determination, may differ among humans.

*KRAS* mutations are considered to occur during initiation or early event in colorectal carcinogenesis [16, 17], but not in the malignant progression of CRC because it has been found in dysplastic lesions and adenomatous polyps, and such mutations alone are insufficient for the sustained growth of cancer. Once the *KRAS* mutations occur, the KRAS activation signaling will be sustained for over 10 years in the somatic evolution of adult cancers. More specifically, the presence of *KRAS* mutations alone is considered to be insufficient for malignant transformations unless they function in cooperation with a particular set of other cancer-related genes in vivo. If this is true, identification of signaling molecules functioning in cooperation with KRAS may allow for the development of a new strategy for suppressing cancer without the use of KRAS inhibitors. MEK inhibitors are being evaluated for their clinical efficacy in targeting CRC with *KRAS* mutations and have a greater dependence on MAPK pathway signaling [18]; however, it seems that MAPK pathway inhibition during the treatment of CRC with *KRAS* mutation remains elusive [6, 19, 20]. Furthermore, studies have shown that MEK inhibitors did not improve overall survival in patients with advanced non-small cell lung cancer (NSCLC) [21] or pancreatic cancer [22] harboring *KRAS* mutations. An effective combination therapy using TBK1 and MEK or BET inhibitors has also been reported in aggressive murine *KRAS*-driven lung cancer [23]. In addition to MEK inhibitors, a recent study revealed that a covalent KRAS inhibitor could inhibit tumor cell growth in NSCLC with *KRAS* G12C mutation [24, 25], but not in CRC [26].

Although many KRAS-associated molecules play an important role in regulating *KRAS* transcription [27], the regulatory mechanisms underlying its activation in vivo have not been fully elucidated. In this study, we first comprehensively analyzed the mutations and expressions of known genes involved in the KRAS signaling pathway in patients with CRC. The *KRAS* G12 mutation is found at a characteristically high frequency and is associated with worse overall survival in patients with CRC [28]. Therefore, next, we explored the potential effector molecules whose gene expression levels differed between CRC patients with wild-type *KRAS* and those with a *KRAS* mutation in codon 12. We then validated these candidate genes by transfecting *KRAS* mutants into human cells. Effective therapies targeting KRAS signaling pathway have not yet been introduced in clinical practice. Moreover, RAS proteins have been dismissed as undruggable targets for many years (5, 6). We hope that this study paves the way for the development of novel treatments that target signaling molecules functioning in the *KRAS* G12-driven CRC.

## Materials and methods

### Subjects

We performed the Whole Exome Sequencing (WES) and Comprehensive Cancer Panel (CCP) using blood samples and fresh surgical specimens. We then conducted Gene Expression Profiling (GEP) using matched tumor and adjacent normal tissues from each patient. Tumor-specific single nucleotide variants (SNVs) were determined by comparing tumor tissue with blood cell data from the same patient. Between January 2014 and January 2017, the samples were obtained from 906 patients with CRC treated with surgery at the Shizuoka Cancer Center Hospital, Shizuoka, Japan (Table 1).

**Table 1:** Characteristics of the colorectal cancer patients

	*KRAS *wild-type	*KRAS *mutated	^a^*P *value
Total number	534	372	
Tumor type			
Colon	302	194	
Rectum	232	178	0.20
Location of the primary tumor			
Anal	3	1	
Ascending	69	76	
Cecum	18	37	
Descending	21	8	
Sigmoid	132	57	
Transverse	59	15	
Rectum	232	178	
Clinical stage			
Stage I	39	52	
Stage II	123	89	
Stage III	303	187	
Stage IV	63	42	
Unknown	6	2	
Age, y			
<45	39	13	
46–55	58	24	
56–65	128	56	
≥66	309	85	
Gender			
Male	337	194	
Female	197	178	0.001
Smoking status			
Nonsmokers	188	165	
Smokers	346	207	0.006
Unknown	0	0	
Pack-years^b^			
0	188	165	
Light smokers (>0 to <20)	90	69	
Heavy smokers (≥20)	237	129	0.079
Smokers but pack-years unknown	19	9	
Unknown	0	0	
Drinking status			
Nondrinkers	118	98	
Drinkers	334	209	0.09
Unknown	82	65	

^a^*P *value by Fisher exact test

^b^Pack-years defined as number of packs of cigarettes smoked per day times of years of smoking

WES/CCP and GEP were performed using the Ion Proton system and Agilent system, respectively. Details of the experimental procedures have been described in previous reports [29–32].

### Ethical statement

All experimental protocols were approved by the Institutional Review Board at the Shizuoka Cancer Center (Authorization Number: 25–33). Written informed consent was obtained from all patients for the participation in this study. All experiments using clinical samples were carried out in accordance with the approved guidelines [33].

### Cell lines

The human 293 embryonic kidney cell line and human CRC cell line, Caco-2, were obtained from the American Type Culture Collection (ATCC; Manassas, VA, USA) and cultured in Dulbecco’s modified Eagle’s medium supplemented with 10% fetal bovine serum at 37 °C in 5% CO_2_. Both 293 and Caco-2 cells have wild-type *KRAS* as well as *BRAF* and *PIK3CA*, which are direct downstream effectors of RAS signaling.

### Construction of KRAS expression vector

To construct the *KRAS* cDNA expression vectors to transduce the entire *KRAS* coding exons representing either the mutant or wild-type forms, the respective cDNA was synthesized using a 1 μg of total RNA isolated from normal breast tissue. The cDNA was amplified using the primers for the *KRAS* sequence including a Kozak translation initiation sequence containing an ATG initiation codon for proper initiation of translation. The polymerase chain reaction (PCR) products were cloned into the pcDNA3.1 D/V5-His vector (Thermo Fisher Scientific) downstream to the human cytomegalovirus promoter to express the KRAS protein fused with a V5-epitope tag at its C-terminus. Site-directed mutagenesis was performed according to the manufacturer’s protocol (In-Fusion HD Cloning Kit, TaKaRa, Japan). The resulting pcDNA3.1D/V5-His/KRAS vectors were designated as pKRAS-WT, pKRAS-A, pKRAS-C, pKRAS-D, pKRAS-R, pKRAS-S, and pKRAS-V, and they harbored wild-type, G12A, G12C, G12D, G12R, G12S, and G12V mutants at codon12 of the *KRAS* cDNA, respectively. A pcDNA3.1 D/V5-His/LacZ (named *pLacZ*) served as a negative control.

### Transfection of KRAS expression vectors into cells

The 293 cells had a high transfection efficiency (90% or more), and the Caco-2 cells were transfected using TransIT-293 transfection reagent (Mirus Bio LLC, Madison) or Lipofectamine 3000 (Invitrogen) and Opti-MEM, as previously described [34]. The cells were seeded at 3–5 × 10^5^ cells/well in 6-well plates; 24–48 h later, when the cells reached 70–80% confluence, they were transfected with pKRAS-WT, pKRAS-A, pKRAS-C, pKRAS-D, pKRAS-R, pKRAS-S, pKRAS-V, or pLacZ expression vector. After 4–5 h, the medium was replaced with DMEM, and the cells were incubated for 24 or 48 h.

### Western blot analyses of transfected cells

Western blot analyses of the cells transfected with either of the vectors indicated above were performed essentially as described [34]. The protein samples were size fractionated using a gradient 12% SDS polyacrylamide gel, and a commercially available antibodies were used for the detection of the V5 peptide tag (Thermo Fisher Scientific) and *β*-actin protein (Sigma Chemical Co, St. Louis, MO).

### Validation of candidate genes using real-time quantitative RT-PCR analysis

A total RNA from cells transfected with pKRAS expression vectors as described above was isolated using Isogen reagent (Nippon Gene, Japan), and the cDNA was synthesized. The cDNA was subjected to the real-time quantitative RT-PCR (qPCR) using the Universal Master Mix according to the manufacturer’s specifications. Primers and TaqMan probes for candidate genes were used along with commercially available online (Thermo Fisher Scientific). The qPCR signal obtained with the optimal cycling parameters for each gene was normalized to *β*-actin.

### Statistical analysis

A significant difference in gene expression between the *KRAS* wild-type and *KRAS*-mutated CRC was calculated using Welch’s t-test, and the significance level was set to 1E-08 by Benjamini-Hochberg (BH) correction for multiple testing. In the comparative analysis of candidate genes, Welch’s *t* test was applied to compare gene expression levels among the vector-transfected cells. Fisher’s exact test was used to compare the subjects between the groups.

## Results

### Whole exome sequencing and deep sequencing of the custom cancer panel in CRC

We used WES to analyse 1074 cancer-related genes from 27 databases [29] in paired tumor tissue and blood samples to detect genetic changes in CRC. Simultaneously, we used the CCP comprising 409 target genes to conduct deep sequencing of tumor tissue samples to validate the WES data. The mean depth of coverage of the target regions was 115-fold for WES and 1027-fold for CCP. *KRAS* mutations were detected in 41.0% of all cases (372/906), which was consistent with the frequencies for these mutations observed in previous studies [35]. The concordance rate between the WES and CCP for *KRAS* mutations was 91.4% (340/372). The non-coincident was composed of WES-negative (15/372) and CCP-negative (17/372) for *KRAS* mutations.

Among *KRAS*-mutated CRC samples, the frequencies *KRAS* mutations were as follows: G12, 64.5% (240/372); G13, 20.2% (75/372); A146, 8.1% (30/372); Q61, 2.7% (10/372); K117, 2.7% (10/372); Q22, 0.5% (2/372); A59, 0.5% (2/372), and 0.3% (1/372) for A14, G77 and Y64 mutations. Within the *KRAS* G12 mutations, the frequencies of the various types of substitutions were 47.9% for *KRAS* G12D (115/240), 26.3% for G12V (63/240), 9.2% for G12A (22/240), 8.8% for G12C (21/240), 5.8% for G12S (14/240), and 2.1% for G12R (5/240).

**Fig. 1:**
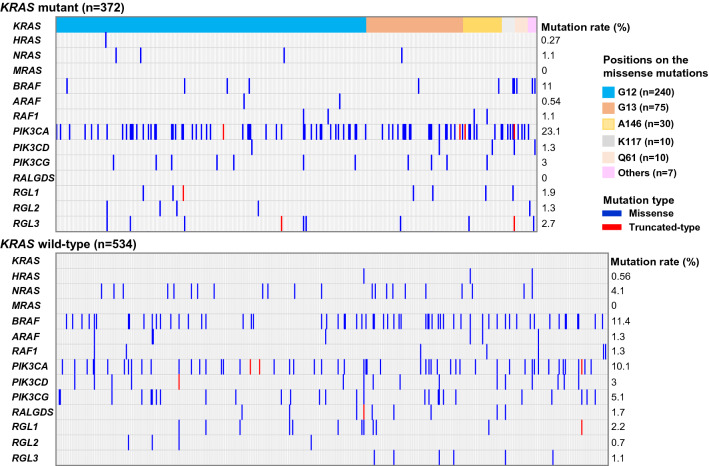
Genomic alterations in the *KRAS*-related genes in CRC. *RAS*-related genes were obtained from the NCI RAS Initiative [6]: **a** Mutation frequencies of genes that directly regulate RAS proteins in 906 colorectal cancer patients with (*n* = 372) or without (*n* = 534) *KRAS* mutations. Each column denotes an individual tumor. Left: percentage of samples with mutations in a given gene. Others (*Pink Square*) in the positions on *KRAS* mutations indicated Q22 (*n* = 2), A59 (*n* = 2), A14 (*n* = 1), G77 (*n* = 1), and Y64 (*n* = 1)

In *KRAS* mutated CRC samples, somatic mutations in *PIK3CA* (86/372, 23.1%) were the most frequently detected among the genes known to mediate RAS-associated responses. On the other hand, somatic mutations in *BRAF* (61/534, 11.4%), *PIK3CG* (27/534, 5.1%), *PIK3CD* (16/534, 3.0%), and *NRAS* (22/534, 4.1%) were frequently detected in *KRAS* wild-type CRC compared to *KRAS*-mutated CRC. The median tumor mutational burden (TMB) in *KRAS* wild-type CRC (*n* = 534) and *KRAS* mutated CRC (*n* = 372) were 8.27 mutations/Mb, and 13.27 mutations/Mb, respectively. Notably, somatic mutations in *RALGDS* were detected in *KRAS* wild-type CRC, but not in *KRAS* mutated CRC. It is intriguing that our WES analysis revealed that the RAS-associated genes were frequently mutated at high levels in patients with *KRAS* wild-type CRC compared to *KRAS*-mutated CRC (Fig. 1).

### Comprehensive gene expression analysis of KRAS pathway-associated genes using DNA microarray

**Fig. 2:**
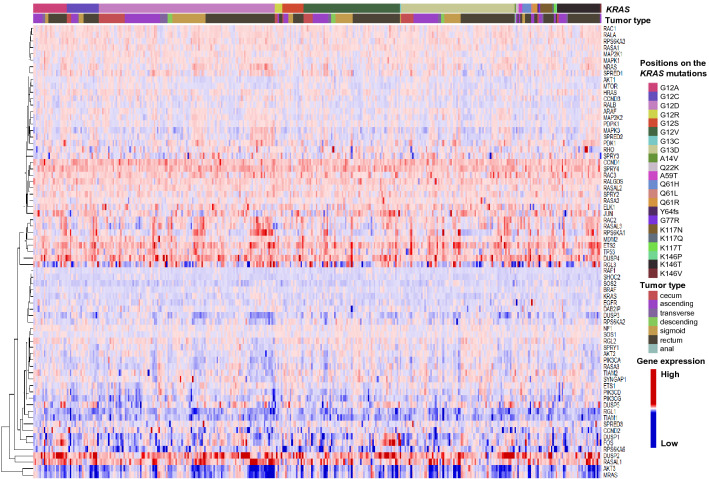
A clustered heat map showing 65 of the *KRAS* pathway-associated genes that are differentially expressed in tumor tissues relative to adjacent normal tissues in 374 CRC with *KRAS* mutations. The tumor type in CRC indicates the location of the primary tumor (right upper panel). The expression levels (log_2_) are normalized for each gene and shown by the graded color scale

**Fig. 3:**
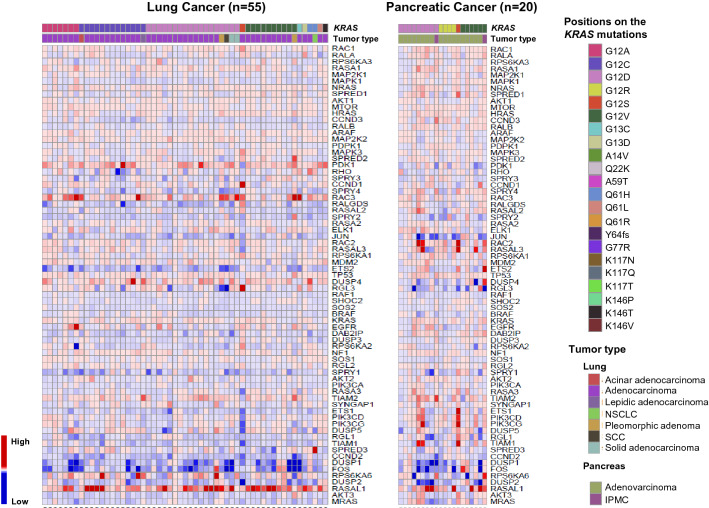
Heat map showing 65 of the *KRAS* pathway-associated genes that are differentially expressed in tumor tissues compared to adjacent normal tissues in 55 lung and 20 pancreatic cancers with *KRAS* mutations. The order of the KRAS-related genes is the same as in CRC samples (Fig. 2). Samples for lung (left) and pancreatic (right) cancers with *KRAS* mutations were obtained from our previous study [29]

Of the known downstream genes in the KRAS pathway, increased expression was observed for *CCND1*, *DUSP2*, *DUSP4*, *ETS2*, *JUN*, *RAC2*, *RAC3*, *SPRY4*, *ELK1, RALGDS*, and *RASAL1* in *KRAS* mutated CRC (Fig. 2). Conversely, the expression levels of *CCND1*, *DUSP2*, *ETS2*, *JUN*, and *RALGDS* were decreased in lung and pancreatic adenocarcinomas with *KRAS* mutations (Fig. 3). The signaling cascades downstream of the KRAS protein leading to the following pathways involving RAF/MAPK/ERK, PI3K/AKT, and RAL GDS/RAL have been well elucidated and are considered to differ according to the tumor type. It is noteworthy that transcription factors, such as *ETS2*, *JUN*, and *ELK1,* were upregulated in the *KRAS* mutated CRC, but not in lung and pancreatic cancers. Thus, the genes corresponding to these transcription factors may be promising targets for treating *KRAS* mutated CRC. However, the differences in expression levels of *ETS2*, *JUN,* and *ELK1* between the *KRAS* mutant and the wild-type were not statistically significant (BH-adjusted *P* value, > 0.26).

### Exploring of the drug-targetable oncogenes functioning with the KRAS-G12 mutant

To exploit the novel *KRAS* G12 mutant targets, GEP was assessed in *KRAS* G12 mutated CRC (*n* = 240) and *KRAS* wild-type (*n* = 390). *KRAS* wild-type CRC harboring mutations in *HRAS*, *NRAS*, *PIK3CA*, *PIK3CD*, *PIK3CG*, *RALGDS*, *RGL1-3*, *BRAF*, *ARAF*, or *RAF1* were excluded from the analysis because mutations in these genes directly affect *KRAS*-mediated signaling. The difference in the normalized signal intensities (fold change, FC) between the tumor and adjacent normal tissues was then calculated. The *KRAS* G12 mutated CRC (*n* = 240) and the selected *KRAS* wild-type CRC (*n* = 390) harbored *APC* mutation at 79.6% (191/240) and 74.9% (292/390), respectively; however, this difference that was not statistically significant (*P* = 0.21). On the other hand, the incidence of *TP53* mutations showed a statistically significant difference (*P* < 0.01) between *KRAS* G12 mutated CRC (64.5%, 143/240) and *KRAS* wild-type CRC (83.1%, 324/390). There were number 13,222 genes that showed a positive FC value (mutant/wild-type) in *KRAS* G12 mutated CRC compared to the *KRAS* wild-type CRC. It was also noted that at least 11 promising candidate molecules showed greater than two-FC between *KRAS* G12 mutant and wild-type and had a BH-adjusted *P* value of less than 1E-08 and showed significant differential expression between these two groups (Table 2).

**Table 2:** List of promising candidate genes that showed significant differences between *KRAS* G12 mutated and wild-type CRC

	Gene	Description	FC_a_	*P *-value	
				Welch’s t test	BH-adjusted
1	*HOXB6*	Homeobox B6	2.72	3.42E-16	4.50E-13
2	*PHLDA1*	Pleckstrin homology like domain family A member 1	3.26	6.02E-15	3.79E-12
3	*BMP4*	Bone morphogenetic Protein 4	2.05	1.22E-14	6.83E-12
4	*OTUB2*	OTU deubiquitinase, ubiquitin aldehydebinding 2	2.81	1.99E-14	1.05E-11
5	*TGFBI*	Transforming growth factor beta 1	2.43	2.92E-14	1.36E-11
6	*SLC28A3*	Solute carrier family 28 member 3	6.78	6.76E-13	1.94E-10
7	*TMEM211*	Transmembrane protein 211	9.95	7.89E-12	1.42E-09
8	*DNAH2*	Dynein axonemal heavy chain 2	4.30	1.33E-11	2.27E-09
9	*FAM169A*	Family with sequence similarity 169 member A	3.36	2.15E-11	3.31E-09
10	*GJB5*	Gap junction protein beta 5	14.06	2.29E-11	3.46E-09
11	*C2orf70 (FAM166C)*	Family with sequence similarity 166 member C	2.66	2.39E-11	3.57E-09

^a^*FC *(Fold Change) in the normalized signal intensities between *KRAS *G12 mutated CRC and *KRAS *wild-type CRC

### Validation of promising candidate genes in KRAS-mediated signaling

**Fig. 4:**
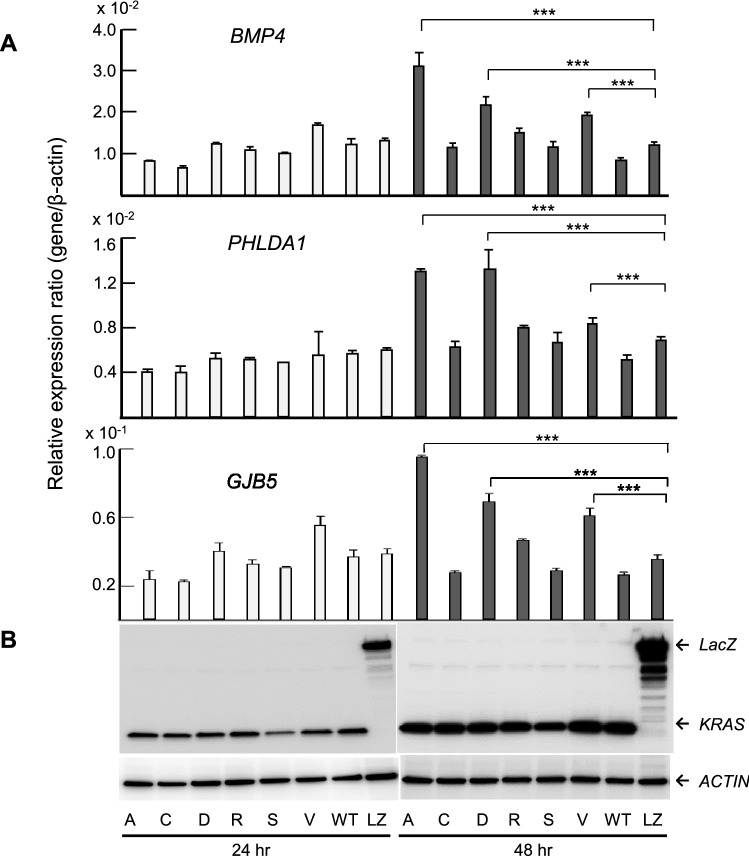
Promising candidate genes are validated using qPCR in the *KRAS* G12 mutant transfected 293 cells: **a** Relative expression ratio is defined as the ratio between the expression level of a gene to that of the internal reference gene, *β*-actin. White and black columns indicate the expression levels at 24 and 48 h after transfection, respectively. The assays are carried out in triplicates and means ± standard deviation are plotted, **b** KRAS protein expression in the 293 cells transfected with *KRAS* mutants, wild-type, or LacZ control vector analyzed using Western blot with V5 and *β*-actin antibodies. The *β*-actin is used as a loading control. A, C, D, R, S, V, WT, and LZ indicate G12A-, G12C-, G12D-, G12R-, G12S-, G12V-, wild-type-, and LacZ transfected cells, respectively. The asterisk indicates ***P value < 0.001

**Fig. 5:**
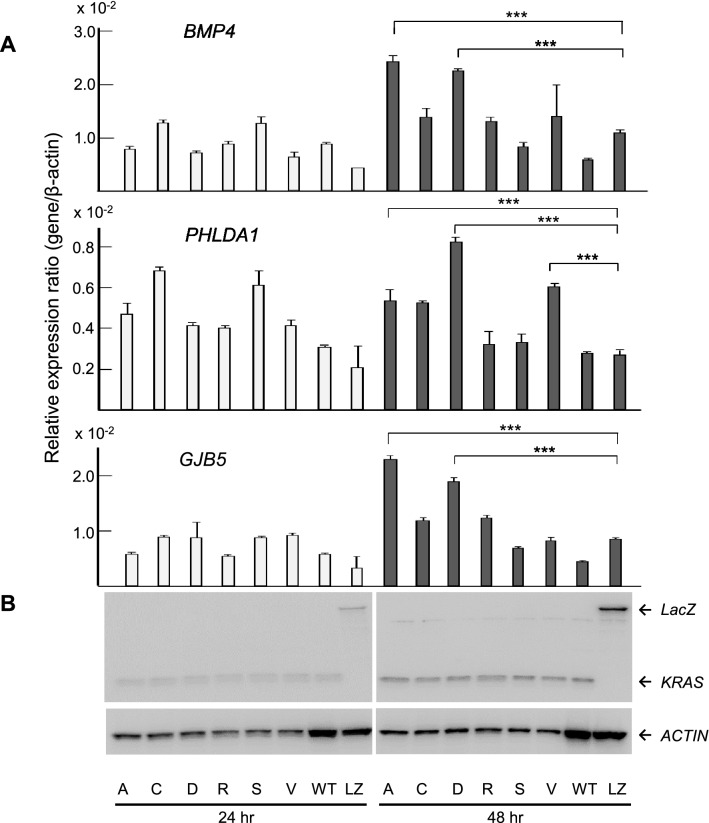
Expression of *BMP4*, *PHLDA1*, and *GJB5* are validated using qPCR in the *KRAS* G12 mutant transfected Caco-2 cells: **a** Relative expression ratio is defined as the ratio between the expression level of a gene to that of the internal reference gene, *β*-actin. **b** KRAS protein expression in the Caco-2 cells are analyzed using Western blot with V5 and *β*-actin antibodies. The assays are carried out the same as that show in Fig. 4. The asterisk indicates ***P value < 0.001

**Fig. 6:**
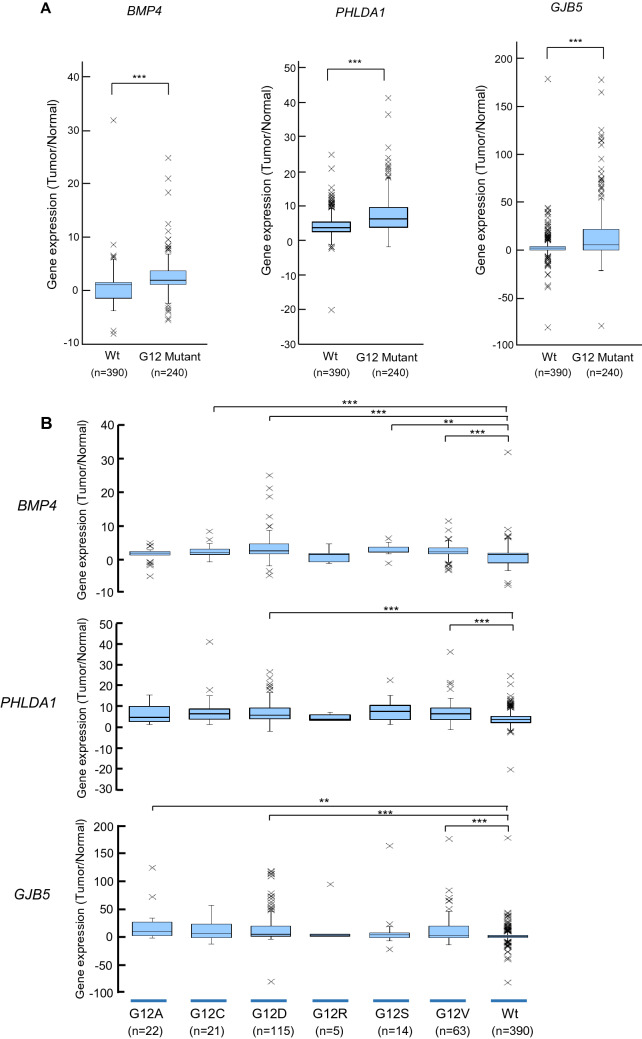
*BMP4*, *PHLDA1*, and *GJB5* expression levels in CRC with *KRAS* G12 mutant and wild-type (**a**) or *KRAS*-G12A, -G12C, -G12D, -G12R, -G12S, -G12V mutants, and wild-type (**b**). The expression level (log2) was normalized for each gene. *** indicates *P* < 0.001; ** indicates *P* < 0.01

**Fig. 7:**
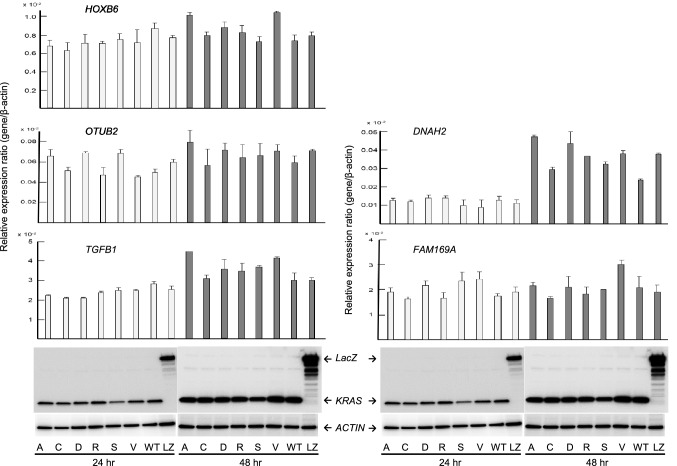
Five genes, excluding *BMP4*, *PHLDA1,* and *GJB5* shown in Table 2 are validated using qPCR in the *KRAS* G12 mutant transfected cell. All genes show a difference in up-regulation but this difference is not significant compared to *KRAS* wild-type or LacZ transfected cells. *SLC28A3*, *TMEM211,* and *C2orf70* genes shown in Table 2 are not detected by qPCR. The assays are carried out in triplicate, and means ± standard deviations are plotted

**Fig. 8:**
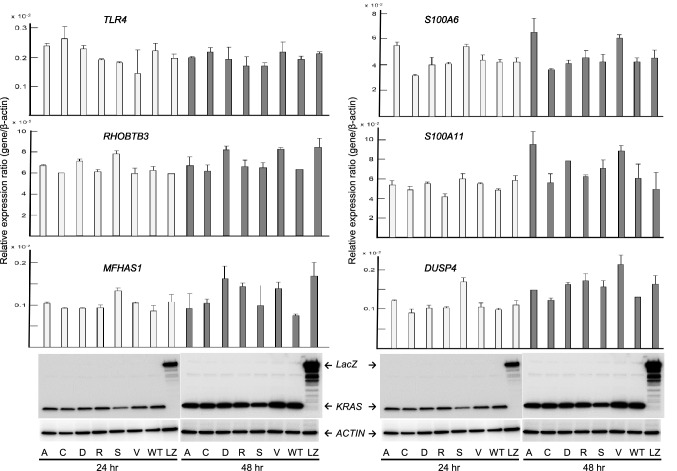
Validation of *TLR4*, *RHOBTB3*, *MFHAS1*, *S100A6*, *S100A11,* and *DUSP4* genes that had a BH-adjusted *P* value less than 1.00E-10 between *KRAS* G12 mutated and wild-type CRC, which have been implicated in the oncogenic function. All genes were not validated by qPCR. The Western blot analysis of transfected cell is the same as that show in Fig. 6. The assays are carried out in triplicate and means ± standard deviation were plotted

To verify the expression levels of the candidate genes in *KRAS* G12 mutated CRC, expression plasmids of *KRAS* variants, designated pKRAS-WT (wild-type), pKRAS-A (G12A), pKRAS-C (G12C), pKRAS-D (G12D), pKRAS-R (G12R), pKRAS-S (G12S), pKRAS-V (G12V), and pLacZ (control vector), were transfected into the human 293 embryonic kidney cells harboring *KRAS* wild-type. The level of gene expression in the transfected cells was analyzed using qPCR. The expression levels of the 11 candidate genes varied depending on the type of *KRAS* mutant, but the expression was effectively induced in G12A, G12D, and G12V mutants. Remarkably, as shown in Fig. 4, *BMP4*, *PHLDA1*, and *GJB5* expression levels were significantly upregulated in the G12A-, G12D-, G12V- transfected cells, compared those in the WT-transfected cells, suggesting that these genes can be added to the list of candidates of *KRAS* G12A, G12D, or G12V target genes in CRC. To re-verify the expression data of *BMP4*, *PHLDA1*, and *GJB5* were validated in the *KRAS* mutants-transfected 293 cells, and real-time RT-PCR analysis was performed for the *KRAS* mutant transfected Caco-2 CRC cells. Although the measured gene expression level was different between the 293 and Caco-2 cells, the effect of *KRAS* mutant transduction, that is, G12D, G12A, and G12V, was confirmed in Caco-2 cells (Fig. 5). This inconsistency in induced gene expression between the 293 and Caco-2 cells may be attributed to differences in transfection efficiency, susceptibility, and cellular differentiation, the nature of which should be explored further. The up-regulation of these genes was re-verified in an independent experiment (data not shown). *BMP4*, *PHLDA1*, and *GJB* expression levels in pairs of tumors and adjacent normal tissues from the patients with CRC obtained using GEP were significantly higher (*P* < 0.001) in the *KRAS* G12 mutant compared with those in the wild-type (Fig. 6a). The *KRAS* G12D and G12V mutants also showed increased expression levels (*P* < 0.001) in comparison with the wild-type (Fig. 6b) Western blot analysis using the V5-tagged antibody showed no difference in the KRAS protein levels between the pKRAS-WT and pKRAS mutated cells. The entire transfection experiment was repeated twice, showing the same KRAS protein level in the transfected cells. The other eight genes (genes shown in Table 2) were not verified by qPCR (Fig. 7). In addition to the 11 candidate genes, we analyzed the *TLR4*, *RHOBTB3*, *MFHAS1*, *S100A6*, *S100A11, and DUSP4* genes that had a BH-adjusted *P* value of less than 1E-09 between *KRAS* G12 mutant and wild-type, but less than two-fold, which have been implicated in the oncogenic functions (Supplementary Table). None of these genes showed a significantly different expression levels in *KRAS* G12 mutant transfected cells from those in the wild-type or LacZ transfected control cells. (Fig. 8).

## Discussion

In this study, we identified *BMP4*, *PHLDA1*, and *GJB5* as the most likely genes that are activated downstream of the *KRAS* G12-driver mutation in CRC, especially the G12A, G12D, and G12V mutations. On the other hand, transfection of the G12C, G12R, and G12S mutants showed lower expression of *BMP4*, *PHLDA1*, and *GJB5,* but higher than those of the wild-type, compared with the G12A, G12D, and G12V mutants. Presently, the detailed mechanism for these differential expression profiles is not clear; however, specific *KRAS* mutations have unique biological and clinical behaviors. Hunter et al. [36] have systemically examined the biochemical and biophysical properties of common *KRAS* mutants and showed that a cell line harboring the G12A mutation, which had high affinity for RAF kinase and low intrinsic GTPase activity, showed the highest sensitivity to MEK inhibitor, suggesting that G12A mutation intensely affects the downstream signal of *KRAS*. In our present study, the highest induction was caused by G12A mutant in several genes (Figs.4 and 5). Additionally, the G12D mutation, which is predicted to be a low RAF activator, is associated with PI3K, but not RAF kinase and does not induce ERK phosphorylation in NIH3T3 cells. G12V, which is predicted to be a moderate RAF activator [36], is associated with both RAF kinase and PI3K in NIH3T3 cells [37]. Therefore, it is suggested that the signals of *KRAS* mutation have different biological properties depending on mutation type and differentially affect the final gene expression process in the signal transduction cascade. The genes identified in our study may be involved in CRC development ant progression by directly or indirectly regulating the expression of these genes, depending on the type of *KRAS* mutation. To clarify the detailed mechanisms of *KRAS* mutation-induced differential gene expression patterns, further investigations are necessary. Furthermore, in CRC, G12A, G12D, and G12V mutations account for 85% of all *KRAS* G12 mutations. Therefore, it may also contribute to the acceleration of personalized medicine for CRC patients with these mutations. Our study has added these genes to the list of those that are possibly involved in colorectal carcinogenesis.

*BMP4* belongs to the TGF*β* superfamily and has been reported to be involved in the regulation of various biological processes such as tissue organization of colonic epithelial cells, interaction between epithelial cells and stromal cells, epithelial-mesenchymal transition (EMT) induction, and metastasis [38, 39]. Additionally, *BMP4* has been reported to promote colon cancer cell invasiveness and tumor formation [40]. Therefore, it is suggested that genes induced by the activation of *BMP4*-dependent signaling may be involved in the carcinogenesis and progression of CRC. In contrast, another study showed that *BMP4* was involved in the suppression of colon cancer cell growth and that the activated *KRAS* down-regulated *BMP4* via the ERK pathway [41]. A possible explanation for this apparent controversy could be that these differential roles accounted to the differences in cell lines used among those studies. Aberrant activation of the Wnt/*β*-catenin pathway enhances *BMP4* signaling in colorectal cancer cells [42]. Therefore, although there was a possibility that *BMP4* expression was increased by inactivation of *APC* in CRC, no difference was observed in the frequency of *APC* mutation depending on the presence or absence of *KRAS* mutations in this study. *PHLDA1* may be a transcriptional activator that is induced by various external stimuli and acts as a mediator of apoptosis, proliferation, differentiation, and cell migration depending on the cell type and physiological context [43]. It has also been suggested that *PHLDA1* is a putative epithelial stem cell marker in the small and large human intestine and contributes to the migration and proliferation of colon cancer cells [44], and it may contribute to the understanding of the oncogenic mechanism of colorectal carcinogenesis. However, the mechanistic basis for *KRAS* activation and/or *PHLDA1* in CRC has not been fully elucidated, and it should be determined by further investigation. *GJB5* is a member of the connexin family that regulates cell adhesion, proteolysis, and motility. Connexins have been shown to function as tumor suppressors in cancer [45, 46] and have been reported to regulate EMT, tumor cell differentiation, and angiogenesis [47]. Among different members of the connexin family, *GJB5* has not been described in association with colorectal cancer or RAS signaling, and the role of *GJB5* in colorectal carcinogenesis remains largely unknown. Therefore, it is prudent to exclude this gene as a drug-targetable candidate in CRC at this time.

In recent years, various combinations of existing molecular targets [48], synthetic lethal partners [49], and immune checkpoint inhibitors [50] for RAS-activating signals have been extensively developed, and tumor suppressive effects have been shown in animal models. The genes identified in this study may be effective targets when used in combination with existing inhibitors of the MAPK pathway, such as MEK or BRAF inhibitors. The role of the genes identified in this study in the carcinogenesis and progression of CRC with *KRAS* G12 mutations may be a modulation of the cancer phenotype, the nature of which should be elucidated in future studies. We believe that our study will lead to further functional characterization of genes in the context of KRAS-based individualized therapy.

## Supplementary Information

Below is the link to the electronic supplementary material.

Supplementary file1 (DOCX 28 KB)
