# Supplementary material for: BMP4 and PHLDA1 are plausible drug-targetable candidate genes for KRAS G12A-, G12D-, and G12V-driven colorectal cancer
Source: Mol Cell Biochem. 2021 May 12;476(9):3469–82. doi: 10.1007/s11010-021-04172-8 (PMC8342352; doi:10.1007/s11010-021-04172-8)
Supplement: Supplementary file 1 — Supplementary file1 (DOCX 28 KB) [file 11010_2021_4172_MOESM1_ESM.docx]

| Supplementary Table 1 | |  |  |
| --- | --- | --- | --- |
| List of genes that showed significant differences (BH-adjusted *P* value <1.00E-8) | | | |
| between *KRAS* G12 mutated CRC and wild-type CRC. | | |  |
|  |  |  |  |
|  |  | *P*-value | |
| Gene | FC | Welch's t test | BH-adjusted |
| *ZNF511* | 1.17 | 9.97E-24 | 2.51E-19 |
| *PPP2R2A* | 1.05 | 2.64E-22 | 3.22E-18 |
| *TAF7* | 0.98 | 3.82E-22 | 3.22E-18 |
| *MFHAS1* | 1.22 | 1.34E-20 | 8.47E-17 |
| *HNRNPA1* | 0.91 | 2.31E-20 | 1.17E-16 |
| *DUSP6* | 1.20 | 1.23E-19 | 5.18E-16 |
| *PXN* | 0.90 | 1.64E-19 | 5.91E-16 |
| *PKM* | 0.93 | 2.78E-18 | 7.02E-15 |
| *CSNK1A1L* | 0.90 | 2.60E-18 | 7.02E-15 |
| *ELP3* | 0.97 | 5.95E-18 | 1.25E-14 |
| *ABHD2* | 1.48 | 2.26E-17 | 4.39E-14 |
| *RASA1* | 0.83 | 3.74E-17 | 6.74E-14 |
| *PLEKHG3* | 0.99 | 5.56E-17 | 8.91E-14 |
| *EPHB4* | 0.85 | 5.66E-17 | 8.91E-14 |
| *RAB10* | 0.85 | 9.06E-17 | 1.34E-13 |
| *BTF3* | 0.89 | 1.42E-16 | 1.98E-13 |
| *HOXB6* | 2.72 | 3.42E-16 | 4.50E-13 |
| *ATP1B1* | 1.55 | 5.93E-16 | 6.55E-13 |
| *PLEKHG3* | 0.95 | 5.98E-16 | 6.55E-13 |
| *CNOT2* | 0.68 | 7.33E-16 | 7.70E-13 |
| *POC1B* | 0.94 | 9.90E-16 | 9.34E-13 |
| *RPS27L* | 0.92 | 1.00E-15 | 9.34E-13 |
| *HNRNPC* | 0.80 | 1.08E-15 | 9.75E-13 |
| *NDFIP1* | 0.80 | 1.46E-15 | 1.27E-12 |
| *PRPF38B* | 0.81 | 1.56E-15 | 1.30E-12 |
| *THG1L* | 0.84 | 1.84E-15 | 1.45E-12 |
| *CTNNBIP1* | 0.93 | 2.85E-15 | 2.12E-12 |
| *INTS10* | 0.91 | 3.18E-15 | 2.23E-12 |
| *SCAF11* | 0.73 | 3.17E-15 | 2.23E-12 |
| *HNRNPA1L2* | 0.71 | 3.83E-15 | 2.61E-12 |
| *PHLDA1* | 3.26 | 6.02E-15 | 3.79E-12 |
| *S100A6* | 1.07 | 5.94E-15 | 3.79E-12 |
| *FPGS* | 0.60 | 6.39E-15 | 3.93E-12 |
| *TNFRSF10B* | 0.91 | 6.83E-15 | 4.10E-12 |
| *HNRNPK* | 0.73 | 7.03E-15 | 4.12E-12 |
| *BMP4* | 2.05 | 1.22E-14 | 6.83E-12 |
| *EPHB4* | 0.74 | 1.60E-14 | 8.79E-12 |
| *NPM1* | 0.77 | 1.83E-14 | 9.82E-12 |
| *OTUB2* | 2.81 | 1.99E-14 | 1.05E-11 |
| *SERPINB6* | 1.01 | 2.22E-14 | 1.12E-11 |
| *RPL41* | 0.80 | 2.60E-14 | 1.24E-11 |
| *TGFBI* | 2.43 | 2.92E-14 | 1.36E-11 |
| *RAB11A* | 0.83 | 4.11E-14 | 1.88E-11 |
| *S100A11* | 1.23 | 4.36E-14 | 1.96E-11 |
| *DUSP4* | 1.63 | 4.87E-14 | 2.16E-11 |
| *EML2* | 1.12 | 4.98E-14 | 2.16E-11 |
| *TRIM16L* | 1.02 | 5.78E-14 | 2.43E-11 |
| *SHB* | 0.86 | 5.69E-14 | 2.43E-11 |
| *CNOT7* | 0.84 | 6.95E-14 | 2.82E-11 |
| *NDUFA12* | 0.77 | 7.05E-14 | 2.82E-11 |
| *SPRY2* | 0.96 | 1.43E-13 | 5.55E-11 |
| *GTF2E2* | 0.84 | 1.82E-13 | 6.77E-11 |
| *HNRNPCL1* | 0.73 | 1.82E-13 | 6.77E-11 |
| *WSB2* | 0.84 | 2.17E-13 | 7.71E-11 |
| *UBR4* | 0.65 | 2.25E-13 | 7.87E-11 |
| *MSH3* | 0.74 | 2.54E-13 | 8.66E-11 |
| *RUFY1* | 0.72 | 2.79E-13 | 9.26E-11 |
| *HNRNPC* | 0.70 | 2.89E-13 | 9.48E-11 |
| *RARS* | 0.65 | 3.00E-13 | 9.71E-11 |
| *ZNF770* | 0.75 | 3.08E-13 | 9.83E-11 |
| *PGAP1* | 1.14 | 3.13E-13 | 9.86E-11 |
| *C4orf36* | 1.29 | 3.92E-13 | 1.18E-10 |
| *PFKP* | 1.00 | 3.86E-13 | 1.18E-10 |
| *LENG9* | 0.71 | 3.91E-13 | 1.18E-10 |
| *SPRED1* | 1.00 | 4.62E-13 | 1.37E-10 |
| *ERI1* | 0.87 | 5.29E-13 | 1.55E-10 |
| *SLC28A3* | 6.78 | 6.76E-13 | 1.94E-10 |
| *NPM1* | 0.74 | 7.14E-13 | 2.02E-10 |
| *CDC42EP1* | 1.00 | 7.27E-13 | 2.04E-10 |
| *INPP1* | 0.95 | 7.40E-13 | 2.05E-10 |
| *ALDOA* | 0.84 | 9.23E-13 | 2.51E-10 |
| *GCN1L1* | 0.59 | 9.51E-13 | 2.55E-10 |
| *ACTR6* | 0.71 | 9.98E-13 | 2.65E-10 |
| *NPM1* | 0.71 | 1.03E-12 | 2.72E-10 |
| *POLR3B* | 0.85 | 1.05E-12 | 2.72E-10 |
| *RUFY1* | 0.70 | 1.06E-12 | 2.72E-10 |
| *NUDT2* | 0.76 | 1.09E-12 | 2.77E-10 |
| *HNRNPA1L2* | 0.65 | 1.31E-12 | 3.30E-10 |
| *FDFT1* | 1.13 | 1.34E-12 | 3.34E-10 |
| *NSA2* | 0.77 | 1.36E-12 | 3.36E-10 |
| *ARHGEF28* | 0.98 | 1.48E-12 | 3.61E-10 |
| *SERPINB6* | 0.92 | 1.50E-12 | 3.61E-10 |
| *RAP1GDS1* | 0.76 | 1.50E-12 | 3.61E-10 |
| *XPO7* | 0.77 | 1.62E-12 | 3.84E-10 |
| *NAA35* | 0.72 | 1.65E-12 | 3.89E-10 |
| *ABLIM1* | 0.96 | 2.11E-12 | 4.89E-10 |
| *UBXN8* | 1.02 | 2.41E-12 | 5.47E-10 |
| *HNRNPA1L2* | 0.65 | 2.40E-12 | 5.47E-10 |
| *PAPD4* | 0.70 | 2.57E-12 | 5.79E-10 |
| *RNF145* | 0.75 | 2.77E-12 | 6.19E-10 |
| *RHOBTB3* | 1.39 | 3.21E-12 | 7.09E-10 |
| *YTHDF2* | 0.64 | 3.31E-12 | 7.25E-10 |
| *TLR4* | 1.27 | 3.43E-12 | 7.39E-10 |
| *LMNA* | 0.89 | 3.50E-12 | 7.48E-10 |
| *MRPS27* | 0.76 | 3.60E-12 | 7.62E-10 |
| *EXOC6* | 0.73 | 3.76E-12 | 7.91E-10 |
| *QDPR* | 0.88 | 3.93E-12 | 8.19E-10 |
| *B3GNTL1* | 0.85 | 4.15E-12 | 8.52E-10 |
| *TRMT10A* | 0.83 | 4.42E-12 | 8.89E-10 |
| *THEM4* | 0.76 | 4.40E-12 | 8.89E-10 |
| *CDC42BPB* | 0.73 | 4.70E-12 | 9.34E-10 |
| *CLDN12* | 0.82 | 4.76E-12 | 9.38E-10 |
| *INF2* | 0.83 | 5.19E-12 | 9.99E-10 |
| *PSMD9* | 0.72 | 5.16E-12 | 9.99E-10 |
| *ARHGEF2* | 0.78 | 5.92E-12 | 1.12E-09 |
| *CARD6* | 1.22 | 6.44E-12 | 1.21E-09 |
| *SRP9* | 0.70 | 6.57E-12 | 1.22E-09 |
| *JMJD7* | 0.77 | 7.03E-12 | 1.29E-09 |
| *NDUFA12* | 0.72 | 7.76E-12 | 1.41E-09 |
| *TMEM211* | 9.95 | 7.89E-12 | 1.42E-09 |
| *CCNG1* | 0.77 | 7.94E-12 | 1.42E-09 |
| *GUK1* | 0.64 | 8.37E-12 | 1.49E-09 |
| *TBX3* | 1.41 | 8.68E-12 | 1.53E-09 |
| *C9orf116* | 1.19 | 1.15E-11 | 2.00E-09 |
| *STK39* | 0.76 | 1.16E-11 | 2.00E-09 |
| *GAPDH* | 0.78 | 1.21E-11 | 2.08E-09 |
| *DNAH2* | 4.30 | 1.33E-11 | 2.27E-09 |
| *PFKP* | 0.91 | 1.39E-11 | 2.35E-09 |
| *SARNP* | 0.70 | 1.47E-11 | 2.45E-09 |
| *ANXA2* | 1.12 | 1.57E-11 | 2.56E-09 |
| *OBFC1* | 0.72 | 1.56E-11 | 2.56E-09 |
| *RPL6* | 0.69 | 1.64E-11 | 2.65E-09 |
| *NDUFA7* | 0.78 | 1.70E-11 | 2.73E-09 |
| *MECR* | 0.70 | 1.71E-11 | 2.73E-09 |
| *LMNA* | 0.85 | 1.79E-11 | 2.84E-09 |
| *FBXO28* | 0.67 | 1.86E-11 | 2.93E-09 |
| *TMCO4* | 0.76 | 2.01E-11 | 3.15E-09 |
| *ZNF524* | 0.65 | 2.06E-11 | 3.21E-09 |
| *EIF4E* | 0.79 | 2.11E-11 | 3.27E-09 |
| *FAM169A* | 3.36 | 2.15E-11 | 3.31E-09 |
| *BLVRB* | 0.68 | 2.22E-11 | 3.39E-09 |
| *CBX5* | 0.68 | 2.23E-11 | 3.39E-09 |
| *GJB5* | 14.06 | 2.29E-11 | 3.46E-09 |
| *LONP2* | 0.71 | 2.33E-11 | 3.50E-09 |
| *C2orf70* | 2.66 | 2.39E-11 | 3.57E-09 |
| *SKP1* | 0.69 | 2.54E-11 | 3.77E-09 |
| *BIN3* | 0.82 | 2.58E-11 | 3.80E-09 |
| *UBQLN1* | 0.59 | 2.76E-11 | 4.03E-09 |
| *RNF34* | 0.64 | 2.81E-11 | 4.07E-09 |
| *AK6* | 0.71 | 3.05E-11 | 4.37E-09 |
| *CLEC2D* | 0.69 | 3.10E-11 | 4.42E-09 |
| *MCPH1* | 0.83 | 3.17E-11 | 4.49E-09 |
| *UBE3C* | 0.67 | 3.21E-11 | 4.52E-09 |
| *HNRNPA1L2* | 0.62 | 3.26E-11 | 4.57E-09 |
| *HOXB2* | 1.09 | 3.53E-11 | 4.92E-09 |
| *CSNK1A1* | 0.60 | 3.60E-11 | 4.99E-09 |
| *PKP4* | 0.53 | 3.76E-11 | 5.15E-09 |
| *MDM2* | 1.00 | 4.07E-11 | 5.52E-09 |
| *STX18* | 0.62 | 4.46E-11 | 6.02E-09 |
| *SRP9* | 0.68 | 4.62E-11 | 6.20E-09 |
| *CCAR2* | 0.76 | 4.66E-11 | 6.22E-09 |
| *CTNNBIP1* | 0.84 | 4.78E-11 | 6.35E-09 |
| *FOXD4* | 1.52 | 4.89E-11 | 6.43E-09 |
| *C1orf21* | 1.07 | 4.89E-11 | 6.43E-09 |
| *WDR36* | 0.69 | 4.92E-11 | 6.43E-09 |
| *CAAP1* | 0.72 | 5.16E-11 | 6.70E-09 |
| *RIOK2* | 0.68 | 5.20E-11 | 6.70E-09 |
| *PACRGL* | 0.68 | 5.45E-11 | 6.90E-09 |
| *CINP* | 0.73 | 5.52E-11 | 6.96E-09 |
| *THEM4* | 0.55 | 5.59E-11 | 7.01E-09 |
| *INTS9* | 0.75 | 5.99E-11 | 7.48E-09 |
| *TCERG1* | 0.58 | 6.54E-11 | 8.13E-09 |
| *ENTPD2* | 1.47 | 7.36E-11 | 9.05E-09 |
| *GUK1* | 0.59 | 7.48E-11 | 9.16E-09 |
| *FAM207A* | 0.60 | 7.65E-11 | 9.32E-09 |
| *NEK6* | 1.00 | 7.69E-11 | 9.33E-09 |
| *FBXO25* | 0.76 | 7.76E-11 | 9.36E-09 |
